# Supplementary material for: Violence and hepatitis C transmission in prison—A modified social ecological model
Source: PLoS One. 2020 Dec 1;15(12):e0243106. doi: 10.1371/journal.pone.0243106 (PMC7707477; doi:10.1371/journal.pone.0243106)
Supplement: S1 COREQ checklist — (DOCX) [file pone.0243106.s001.docx]

**Consolidated criteria for reporting qualitative studies (COREQ): 32-item checklist**

Developed from:

Tong A, Sainsbury P, Craig J. Consolidated criteria for reporting qualitative research (COREQ): a 32-item checklist for interviews and focus groups. *International Journal for Quality in Health Care*. 2007. Volume 19, Number 6: pp. 349 – 357

**YOU MUST PROVIDE A RESPONSE FOR ALL ITEMS. ENTER N/A IF NOT APPLICABLE**

| **No. Item** | **Guide questions/description** | **Response** | **Reported on Page #** |
| --- | --- | --- | --- |
| **Domain 1: Research team and reﬂexivity** |  |  |  |
| *Personal Characteristics* |  |  |  |
| 1. Inter viewer/facilitator | Which author/s conducted the interview or focus group? | Luke McCredie | Title page |
| 2. Credentials | What were the researcher’s credentials? E.g. PhD, MD | Hossain M S Sazzad, MBBS, MHE, PhD candidate, Kirby Institute, UNSW Sydney  Luke McCredie, Registered Nurse, Research Nurse, Centre for Health Research in Criminal Justice, Justice Health  Carla Treloar, PhD, Scientia Professor and Director, Centre for Social Research and Health, UNSW Sydney  Andrew R Lloyd, MBBS, MD, FRACP, PhD, Professor, Kirby Institute, UNSW Sydney  Lise Lafferty, PhD Research Fellow, Kirby Institute and Centre for Social Research in Health, UNSW Sydney | Title page |
| 3. Occupation | What was their occupation at the time of the study? | At the time of data collection, Luke McCredie was a registered nurse employed by the Principal Investigator Andrew Lloyd for longitudinal cohort study in prison | N/A |
| 4. Gender | Was the researcher male or female? | LM - Male | N/A |
| 5. Experience and training | What experience or training did the researcher have? | LM has been a nurse with Justice Health & Forensic Mental Health Network NSW for more than 10 years. He is trained in psychology.  HS is currently undertaking a PhD in which he is utilizing data from the HITS-P study to explore non-injecting risk factors associated with hepatitis C transmission in the prison setting. He is supervised by AL and LL. AL has over two decades experience treating hepatitis C in the prisoner population. LL is a social scientist with several years’ experience conducting interviews in the prison setting and analyzing qualitative data. CT supervised the qualitative data collection of the HITS-P study. | Methods (Page 5) |
| *Relationship with participants* |  |  |  |
| 6. Relationship established | Was a relationship established prior to study commencement? | LM had been a nurse with Justice Health & Forensic Mental Health Network NSW, was engaged in HITS-p study for 10 years and collected blood and conducted behavioral survey before starting the qualitative data collection. | Methods (Page 5) |
| 7. Participant knowledge of the interviewer | What did the participants know about the researcher? e.g. personal goals, reasons for doing the research | The participants knew LM since starting their enrolment in HITS-p cohort. Participants were well informed about the personal goal and reason of the qualitative study during study participation. He is trained in psychology. | Methods (Page 5) |
| 8. Interviewer characteristics | What characteristics were reported about the inter viewer/facilitator? e.g. Bias, assumptions, reasons and interests in the research topic | LM was well trained and supervised by the HITS-p study investigators including CT who was an experienced qualitative social researcher. | Methods (Page 5) |
| **Domain 2: study design** |  |  |  |
| *Theoretical framework* |  |  |  |
| 9. Methodological orientation and Theory | What methodological orientation was stated to underpin the study? e.g. grounded theory, discourse analysis, ethnography, phenomenology, content analysis | The analysis was informed by both a deductive and inductive approach.  Each aspect of the thematic analysis, that is, the interpretations and meanings drawn from the interview data was critically examined and summarised along with supporting quotes. | Methods (Page 7) |
| *Participant selection* |  |  |  |
| 10. Sampling | How were participants selected? e.g. purposive, convenience, consecutive, snowball | All participants were selected purposively to represent injecting/non-injecting risk exposes and with /without HCV infected prison population. | Methods (Page 5) |
| 11. Method of approach | How were participants approached? e.g. face-to-face, telephone, mail, email | LM informed the prisoners about the qualitative study and offered the opportunity to participate. Then the nurse explained the purpose of the study and the prisoner’s right to accept or decline the offer (a decision to not participate in the qualitative component had no bearing on their involvement in the larger HITS-p study or their relationships with Corrective Services NSW or Justice Health & Forensic Mental Health Network). | Methods (Page 5) |
| 12. Sample size | How many participants were in the study? | Twenty-three people in prison participated in this study, eight of whom were female. Of this group, 10 had no detectable HCV RNA (not exposed to HCV) at the time of interview, 5 had chronic HCV infection (persistent infection) and 8 had recent HCV infection in prison. | Results (Page 7) |
| 13. Non-participation | How many people refused to participate or dropped out? Reasons? | None | N/A |
| *Setting* |  |  |  |
| 14. Setting of data collection | Where was the data collected? e.g. home, clinic, workplace | The data were collected by independent health care provider in a clinic room in prison setting in absence of any correctional officer. | Methods (Page 6) |
| 15. Presence of non-participants | Was anyone else present besides the participants and researchers? | No | N/A |
| 16. Description of sample | What are the important characteristics of the sample? e.g. demographic data, date | Twenty-three people in prison participated in this study, eight of whom were female. Of this group, 10 had no detectable HCV RNA (not exposed to HCV) at the time of interview, 5 had chronic HCV infection (persistent infection) and 8 had recent HCV infection in prison. | Results (Page 7) |
| *Data collection* |  |  |  |
| 17. Interview guide | Were questions, prompts, guides provided by the authors? Was it pilot tested? | Interview guide was prepared, and it was rigorously discussed among the authors. Probes were used to facilitate discussions. | Methods (Page 6) |
| 18. Repeat interviews | Were repeat inter views carried out? If yes, how many? | No | N/A |
| 19. Audio/visual recording | Did the research use audio or visual recording to collect the data? | Interviews were audio recorded. | Methods (Page 6) |
| 20. Field notes | Were ﬁeld notes made during and/or after the interview or focus group? | Verbal debriefing was done between LM and CT | Methods (Page 5) |
| 21. Duration | What was the duration of the inter views or focus group? | The duration of the interviews ranged from 30 to 70 minutes. | Methods (Page 6) |
| 22. Data saturation | Was data saturation discussed? | The data were collected until similar response were coming following subsequent interviews. We found repeated findings from the responses of 23 participants and did not increase the number of interviews to ensure we did not overburden this vulnerable population. | Methods (Page 7) |
| 23. Transcripts returned | Were transcripts returned to participants for comment and/or correction? | No | N/A |
| **Domain 3: analysis and ﬁndings** |  |  |  |
| *Data analysis* |  |  |  |
| 24. Number of data coders | How many data coders coded the data? | Two authors (CT and HS) coded the data. | Methods (Page 7) |
| 25. Description of the coding tree | Did authors provide a description of the coding tree? | Coding framework was discussed among the authors. CT and LM developed the primary coding with multiple sub-coding. Subsequently HS developed secondary coding to develop the modified social ecological model. LL conducted secondary coding of random samples to ensure consistency. | Methods (Page 7) |
| 26. Derivation of themes | Were themes identiﬁed in advance or derived from the data? | Themes were derived from the data. However, the main goal of the study was to utilize data in the modified social ecological framework, which informed the structure of the themes. | Methods (Page 6, 7) |
| 27. Software | What software, if applicable, was used to manage the data? | NVivo software, Microsoft Word and Microsoft Excel | Methods (Page 7) |
| 28. Participant checking | Did participants provide feedback on the ﬁndings? | No | N/A |
| *Reporting* |  |  |  |
| 29. Quotations presented | Were participant quotations presented to illustrate the themes/ﬁndings? Was each quotation identiﬁed? e.g. participant number | Yes, quotations were presented to illustrate the themes/findings, and each quotation was identified with an anonymous participant identifier. | Results (Page 7 - 11) |
| 30. Data and ﬁndings consistent | Was there consistency between the data presented and the ﬁndings? | Yes, there was consistency between the data presented and the findings. | Discussion (Page 16) |
| 31. Clarity of major themes | Were major themes clearly presented in the ﬁndings? | Yes, major themes were clearly presented in the Results section using specific sections regarding each theme. | Results (Page 7 - 11) |
| 32. Clarity of minor themes | Is there a description of diverse cases or discussion of minor themes? | No | N/A |

**Once you have completed this checklist, please save a copy and upload it as part of your submission. When requested to do so as part of the upload process, please select the file type: *Checklist*. You will NOT be able to proceed with submission unless the checklist has been uploaded. Please DO NOT** **include this checklist as part of the main manuscript document. It must be uploaded as a separate file.**
