# Supplementary material for: Violence and hepatitis C transmission in prison—A modified social ecological model
Source: PLoS One. 2020 Dec 1;15(12):e0243106. doi: 10.1371/journal.pone.0243106 (PMC7707477; doi:10.1371/journal.pone.0243106)
Supplement: S1 Appendix — (DOCX) [file pone.0243106.s002.docx]

**Appendix 1: Interview schedule – Violence part:**

(Note this is a complete list of topics to be covered, this list is to ensure that the concepts behind the issues are clear to the interviewer. Issues for discussion will be raised with the participant in conversational style)

Opening

Thank you for agreeing to be part of this interview

This conversation is focused on issues associated with hepatitis C.

You don’t have to answer any questions that you feel uncomfortable about.

Your name will not be recorded anywhere. We will change any information that identifies you or anyone else

*Emphasise no names needed – and that information should not be provided by participant that would implicate themselves or others in serious illegal activity*

Fighting/Violence

What type of violence have you seen in prison?

What are the situations in which violence occurs?

What are the things that people worry about violence - ie possible outcomes -

Does hepatitis C figure in this?

Demographics

- Age
- Age at first injection
- Length of current sentence
- Is this a first sentence?
- Location (prison)

Closing

Would you like to find out more about hepatitis C?

- If yes, make referral

Thank you for your time

Remind re confidentiality protections. Report will be written in *Users News.*
