# Supplementary material for: Violence and hepatitis C transmission in prison—A modified social ecological model
Source: PLoS One. 2020 Dec 1;15(12):e0243106. doi: 10.1371/journal.pone.0243106 (PMC7707477; doi:10.1371/journal.pone.0243106)
Supplement: Attachment — Submitted filename: HCV Australia Review.docx [file pone.0243106.s003.docx]

PLOS One Review

Comments to authors

This is a well-written and organized manuscript presenting new and policy-relevant qualitative data about how prisoners understand and navigate the risks of contracting and living with HCV. Overall, the application of a modified social-ecological model (MSEM) to framing and analyzing the risks articulated by prisoners in qualitative interviews works as a solid frame for the piece. My critiques and suggestions for revisions center around (1) the way the methods were described and the need to engage more with why certain choices were made, (2) wanting a bit more information about the context of the larger study of which these qualitative interviews were a sub-study, (3) wanting to understand a bit more why the focus in this study was on violence (as opposed to other common alternatives like drug use and tattooing) as a means of HCV transmission, and (4) hoping the authors will engage with the actual or potential role of institutional actors (prison staff, bureaucrats) in transmission. I’ll discuss each in turn below.

First, I had questions about methods throughout, starting with the answers to the standard PLOS questions about data.

- The authors note that data is available for “researchers who meet the criteria for access to confidential data.” What does this mean? It sounds like it means it is practically only available to the researchers. I certainly hope not just anyone can approach a human research ethics committee and meet requirements for access to confidential data. If this is qualitative data about a vulnerable population, there may well be reasons to prevent making it available, distinguishing it from other data sources that might be more readily shared. One alternative possibility could be providing a list of quotations used in analysis as an appendix. At any rate, the authors need to be clear about what is and is not available, why, and what the alternatives are.
- Next, the authors note that a nurse assisting with the larger study about HCV of which this was a sub-study both recruited and interviewed participants. They also note written, informed consent was obtained, and participants received $10. Why was a nurse chosen for recruitment and interviews; were concerns about biased engagement and conflicts of interest raised and addressed, either in the process of making this methodological choice, or in the process of recruiting and conducting the interviews? (The conflict I imagine: a medical provider switching roles into researcher, obtaining much more personal information than usual or necessary from a patient, and being potentially biased by that information, especially if they learn a patient is violent or ignoring health recommendations; likewise, a patient might not be fully open with a medical provider about risky activities.)
- Also in terms of the structure of the study, why was written consent obtained, and will that written consent be maintained? At least in the United States, prison studies often obtain waivers of written consent to maximize anonymity and mitigate any risk of individuals being identified and persecuted for anything they might have said.
- In terms of the participation incentive of AU$10 – how valuable is this to prisoner participants? In the United States, that could buy basic hygiene items, a few phone calls home, etc., and would be very meaningful – perhaps even too meaningful; I would like to see a discussion of how this amount was reached, and an analysis of how it struck the appropriate balance between crediting participants for their time without unduly incentivizing them to participate.
- How closed or open-ended was the interview guide the authors used? More detail about its structure (as opposed to just its substance) might be valuable, perhaps even it could be included in an appendix?
- On page 6, line 123, in the methods section, the authors use the term PWID without spelling it out.
- In their description of their analysis of the data, the authors say the transcripts were “read closely,” organized with a “coding frame,” and then re-coded based on the five layers of the MSEM. I have many questions. First of all, what happened between close reading and the coding frame? Were memos written? Were transcripts coded and re-coded, and was any interrater reliability established? How many codes were generated? Were they consolidated in some way? How did the codes relate to the MSEM? Was the MSEM something the authors had planned to apply from the beginning, structuring their interview questions and analysis, or was it something that emerged for the data? Was there any relationship between the coding frame and the MSEM, or were these separate projects, with other analyses of the interviews planned for other purposes?
- Given the very small number of participants in this study, are the authors confident that the level of identifying detail they provide will not compromise anonymity? Providing a table of characteristics of participants (how many of each gender, age, other demographics collected, and how many fell into each category of risk behavior) could alleviate this concern, especially if there are a large enough number of people in each category (often 10, but, here 5 would seem reasonable).
- How many people were recruited for this study? How many refused?

Second, there was a major gap, to my mind, in the presentation of the background and methods, relating to the relationship between this sub-study, and the larger study of which it was a part. The authors describe the ten-year period of the larger study, but give little context about numbers recruited and/or the scale of the larger study, or even their own study. How many participants were recruited for the qualitative interviews? How many refused? Was 23 the target number, or were the authors hoping for more and faced some challenges in recruitment? How many prisoners participated in the larger, 10-year study? Was the qualitative study always envisioned as part of the larger study, or did it grow out of something learned over the course of the larger study? Generally, better situating this study would help to clarify methods, the relevance and representativeness of the findings, and also contextualize findings.

Third, the contextualization of this study in existing literatures was a bit light, making the focus seem more narrow than necessarily does the work justice. In some cases this “lightness” of literature came out in over-simplifications, e.g., “Due to the criminalization of drug use, people in prison have higher prevalence of HCV infection than those in the community” (page 3, lines 36-37). This is a strong claim simplifying multiple complex mechanisms of inequality and disparate treatment institutionalized in incarceration. First of all, are there not many reasons why people might come into prison with higher rates of HCV infection (all kinds of risk factors pre-prison beyond being criminalized drug users)? In fact, stating that drug use explains all HCV infection in a given population (like prisoners) runs the risk of implying that everyone with HCV is a drug user, a troubling implication. Secondly, are there not many reasons (as described in this article) why people in prison might subsequently incur higher rates of HCV infection, many more structural than individual, as this statement implies; e.g., abuse at the hands of other prisoners or staff, lack of adequate healthcare, lack of knowledge of risks, etc. In other cases, the “lightness” of the literature review came out in the focus of the piece on physical violence in prison as a means of HCV transmission. Can the authors elaborate, beyond the fact that prison violence has not been well-studied as a mechanism of HCV transmission, on why they focused on violence to the exclusion of other modes of transmission? Did they always intend to focus on violence and ask only about this in interviews? Was there some evidence from the larger study about the relevance of violence for transmission? Is there some sense that violence may be closely tied to other means of transmission? This is implied in the “2. Network level” findings section, but could be much further explored. For instance, are tattoos linked to violence (they certainly are associated by prison officials with gang membership in the United States, and prisoners often get them for protective reasons)? And/or is there some sense that public health interventions to mitigate HCV transmission in violent encounters might be beneficial in other ways in the prison environment?

Fourth, the underlying frame of this study seemed to be focused on prisoners as individuals, making somewhat free (if constrained) choices about exposing themselves to HCV and managing that exposure. This belies the reality of incarceration as an institution frequently so oppressive that free choices are more than constrained. Perhaps this is an American perspective, and Australian researchers might dis-abuse me of this and convince me Australian prisons are different, though I have read little to convince me of that. A few specific points along these lines: In American prisons, I have never heard of prisoners thinking about or fearing HCV transmission through violent interactions, but every correctional officer I know fears being infected with HCV by prisoners who spit at or otherwise attack them. Yet I know of now such cases of transmission in that manner. This disconnect seems important to address in this piece: how often is HCV transmitted among prisoners through violent interactions? Is this just a staff fear being operationalized in this study? Why were staff not included in this kind of analysis? Might they be included in future analyses? Relatedly, on pages 18-19, the authors discuss the possibility and pros and cons of ethnic clustering in prison, again writing as if prisoners are in control of ethnic tensions, when, in fact, much research (again in the United States, though I cannot imagine there are not comparable analyses of Aboriginal tensions in Australia) has documented how correctional staff leverage innate ethnic differences to encourage and amplify racial tensions, as a means of turning prisoners against each other rather than against their keepers.

One additional minor note – page 14, lines 36-36: The discussion of lock-down/segregation felt out of place and disconnected from the rest of the analysis, though potentially fruitful to explore and better integrate. Though I have reviewed this piece with an eye for details and frameworks that need to be clarified, the study seems valuable and like an important contribution to the literature, and I would look forward to reading a revision.
